# Antifungal Activity of Resveratrol against *Botrytis cinerea* Is Improved Using 2-Furyl Derivatives

**DOI:** 10.1371/journal.pone.0025421

**Published:** 2011-10-11

**Authors:** Francesco Caruso, Leonora Mendoza, Paulo Castro, Milena Cotoras, Maria Aguirre, Betty Matsuhiro, Mauricio Isaacs, Miriam Rossi, Angela Viglianti, Roberto Antonioletti

**Affiliations:** 1 Istituto di Chimica Biomolecolare, Consiglio Nazionale delle Ricerche, University of Rome, Istituto Chimico, Rome, Italy; 2 Facultad de Química y Biología, Universidad de Santiago de Chile, Santiago, Chile; 3 Facultad de Ciencias, Departamento de Química, Universidad de Chile, Santiago, Chile; 4 Vassar College, Department of Chemistry, Poughkeepsie, New York, United States of America; 5 University of Rome, Istituto Chimico, Rome, Italy; University of Sydney, Australia

## Abstract

The antifungal effect of three furyl compounds closely related to resveratrol, (E)-3,4,5-trimethoxy-β-(2-furyl)-styrene (**1**), (E)-4-methoxy-β-(2-furyl)-styrene (**2**) and (E)-3,5-dimethoxy-β-(2-furyl)-styrene (**3**) against *Botrytis cinerea* was analyzed. The inhibitory effect, at 100 µg ml^−1^ of compounds **1**, **2**, **3** and resveratrol on conidia germination, was determined to be about 70%, while at the same concentration pterostilbene (a dimethoxyl derivative of resveratrol) produced complete inhibition. The title compounds were more fungitoxic towards *in vitro* mycelial growth than resveratrol and pterostilbene. Compound **3** was the most active and a potential explanation of this feature is given using density functional theory (DFT) calculations on the demethoxylation/demethylation process. Compound **3** was further evaluated for its effects on laccase production, oxygen consumption and membrane integrity of *B. cinerea*. An increase of the laccase activity was observed in the presence of compound **3** and, using Sytox Green nucleic acid stain, it was demonstrated that this compound altered *B. cinerea* membrane. Finally, compound **3** partially affected conidia respiration.

## Introduction

*Botrytis cinerea* is a facultative phytopathogenic fungus that attacks flowers, fruits, leaves, and stems of more than 200 plant species causing several pre- and post-harvest diseases [1]. The continuous use of commercial fungicides such as dicarboximide and benzimidazole has caused the appearance of highly resistant strains of *B. cinerea* and the contamination of soil and water [2]. However, some natural products isolated from plants exert antifungal activity and could be good alternatives to commercial fungicides [3]. Some plant secondary metabolites, stilbenes, have received considerable interest in this area also because of their role in human health [4].

The resistance of *Vitis vinifera* grape to infection by *B. cinerea* is due, in part, to the plant response through production of several stilbenes including resveratrol, pterostilbene and ε-viniferin, a resveratrol dehydrodimer [5]. These compounds have been tested *in vitro* against *B. cinerea* and results show that pterostilbene inhibits conidia germination and *in vitro* mycelia growth of *B. cinerea* more efficiently than resveratrol, indicating that methylation of hydroxyphenyl groups is important to the antifungal activity [5]. Schouten *et al.* demonstrated that resveratrol, while not toxic to *B. cinerea*, is converted into a fungitoxic compound by a specific laccase of this fungus that then causes self-intoxication [6]. Laccases are copper-containing polyphenol oxidases that catalyze the oxidation of phenolic compounds and the reduction of molecular oxygen into water [7].

The mechanism of action of these stilbenes against *B.cinerea* is not well understood. It has been suggested that resveratrol inhibits the respiration of fungal cells, probably by acting as an uncoupling agent [8]. Another explanation for the mode of action of hydroxystilbenes may involve membrane peroxidation [9]. Pterosilbene causes destruction of the endoplasmic reticulum and the nuclear and mitochondrial membranes in *B. cinerea* dormant conidia [10]. A positive correlation among antifungal activity of natural and synthetic stilbenes and their hydrophobicity was found [11] suggesting that pterostilbene is more active than the less hydrophobic resveratrol, due to its increased diffusion through the cytoplasmic membrane.

*In vitro* evidence suggests that pathogenicity of *B. cinerea* strains in grapevines is associated with their ability to degrade pterostilbene and resveratrol [12]; those that metabolize stilbenes by a laccase-like stilbene oxidase [12]–[13] were more pathogenic to the grapevine than those that do not. Of three different *B. cinerea* laccase genes that have been characterized, *Bclcc*1, *Bclcc*2 and *Bclcc*3, resveratrol induces only *Bclcc*2 expression in liquid culture, suggesting that this gene participates in the metabolic oxidation of stilbene derivatives [6].

Since co-planarity between rings and the connecting double bond in stilbene based compounds, like resveratrol, seems to be a central feature for other biological activities [14]–[15], we were interested in exploring whether this feature contributed to the antifungal mechanism by choosing three coplanar furyl derivatives of resveratrol. In this study, we also explore the antifungal activity against *B. cinerea* isolate T50 of stilbene derivatives having increasing number of methoxy groups since methoxy group substitution has a marked influence on biological properties [16]–[18]. Density functional theory (DFT) calculations were used to analyze the fungitoxic structure-activity relationship of these compounds. The effect of the most active furyl resveratrol derivative on laccase production, oxygen consumption and membrane integrity was also evaluated.

## Results and Discussion

### Antifungal activity characterization

**Figure 1:**
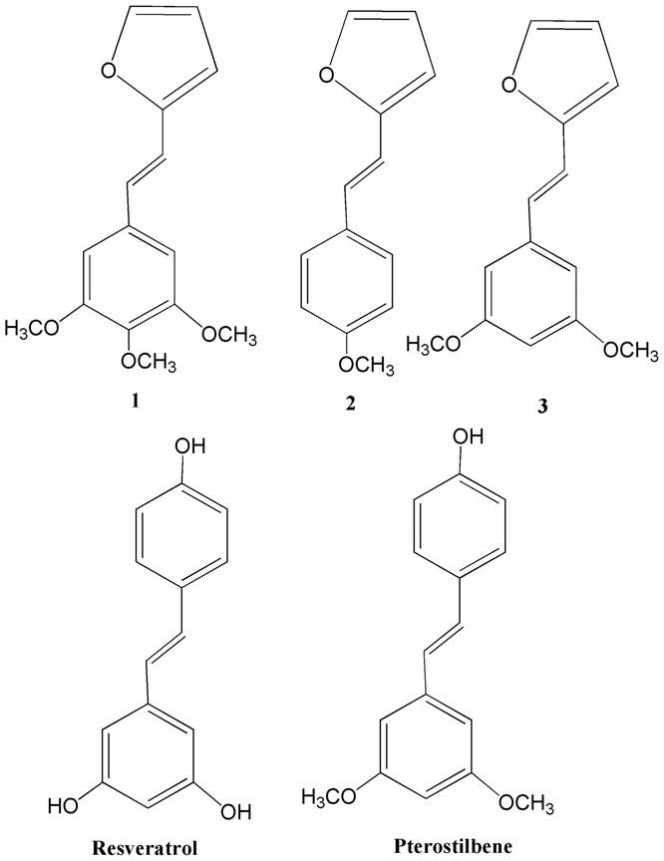
Compounds used in this study. **1**: (E)-3,4,5-trimethoxy-β-(2-furyl)-styrene, **2**: (E)-4-methoxy-β-(2-furyl)-styrene, **3**: (E)-3,5-dimethoxy-β-(2-furyl)-styrene. Resveratrol and pterostilbene were utilized as control.

**Figure 2:**
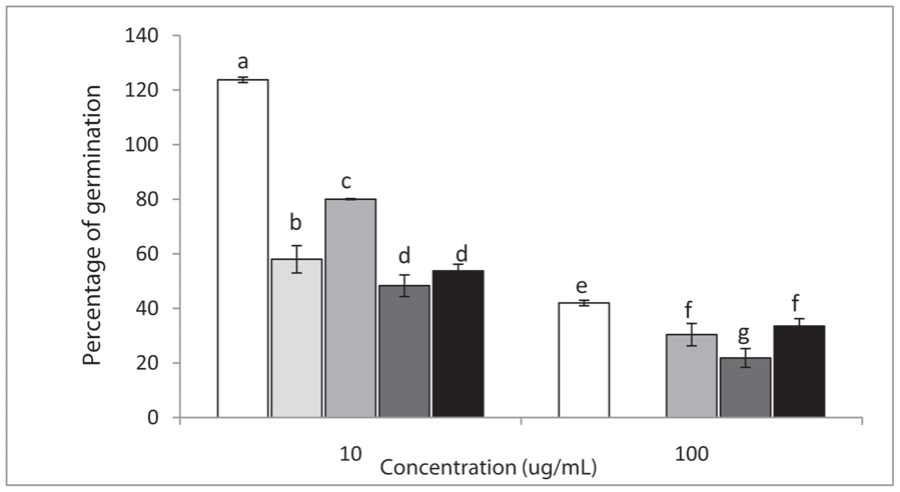
Effect of the compounds on conidial germination of *B. cinerea*. From left to right: Resveratrol, pterostilbene, compound **1**, compound **2** and compound **3**, see also “Materials and methods section k Procedure information about figures”. Different letters indicate that the means are significantly different at P<0.05.

Figure 1 shows the compounds used to determine antifungal activity against *B. cinerea*. Resveratrol and pterostilbene were used as controls. The effect of these compounds on *in vitro* mycelia growth in solid media was determined after 96 hours of incubation (Table 1). Compounds **1**, **2**, and **3** were more active than the control compounds and compound **3** with two methoxy groups had the highest antifungal activity. In addition, the biological effect of these compounds on the conidia germination of *B. cinerea* was evaluated after 7 h of incubation (Figure 2). At 100 µg ml^−1^, pterostilbene completely inhibited conidia germination and the other compounds showed about 70% inhibition. At a lower concentration, 10 µg ml^−1^, resveratrol stimulated germination, compounds **2** and **3** and pterostilbene showed about 50% inhibition and compound **1** showed about 20% inhibition. The title compounds did not produce morphological changes in the germ tube (data not shown).

**Table 1:** Effect of compounds on *in vitro* mycelial growth of *B. cinerea*.

Compounds	Log*P* a	ED_50_ b±SD(µg ml^−1^)
Compound 1	4.0	71.6±4.5
Compound 2	4.4	42.0±5.4
Compound 3	4.3	16.6±3.1
Resveratrol	3.0	350±6.9
Pterostilbene	4.1	100±3.7

a Estimated lipophilicity values and sigma, calculated using Advanced Chemistry Development (ACD/Labs) Software V9.04 (1994–2010 ACD/Labs), as stored in SciFinder.

b Determination of median effective doses (ED_50_), based on colony diameter measurements after 96 h of incubation.

**Figure 3:**
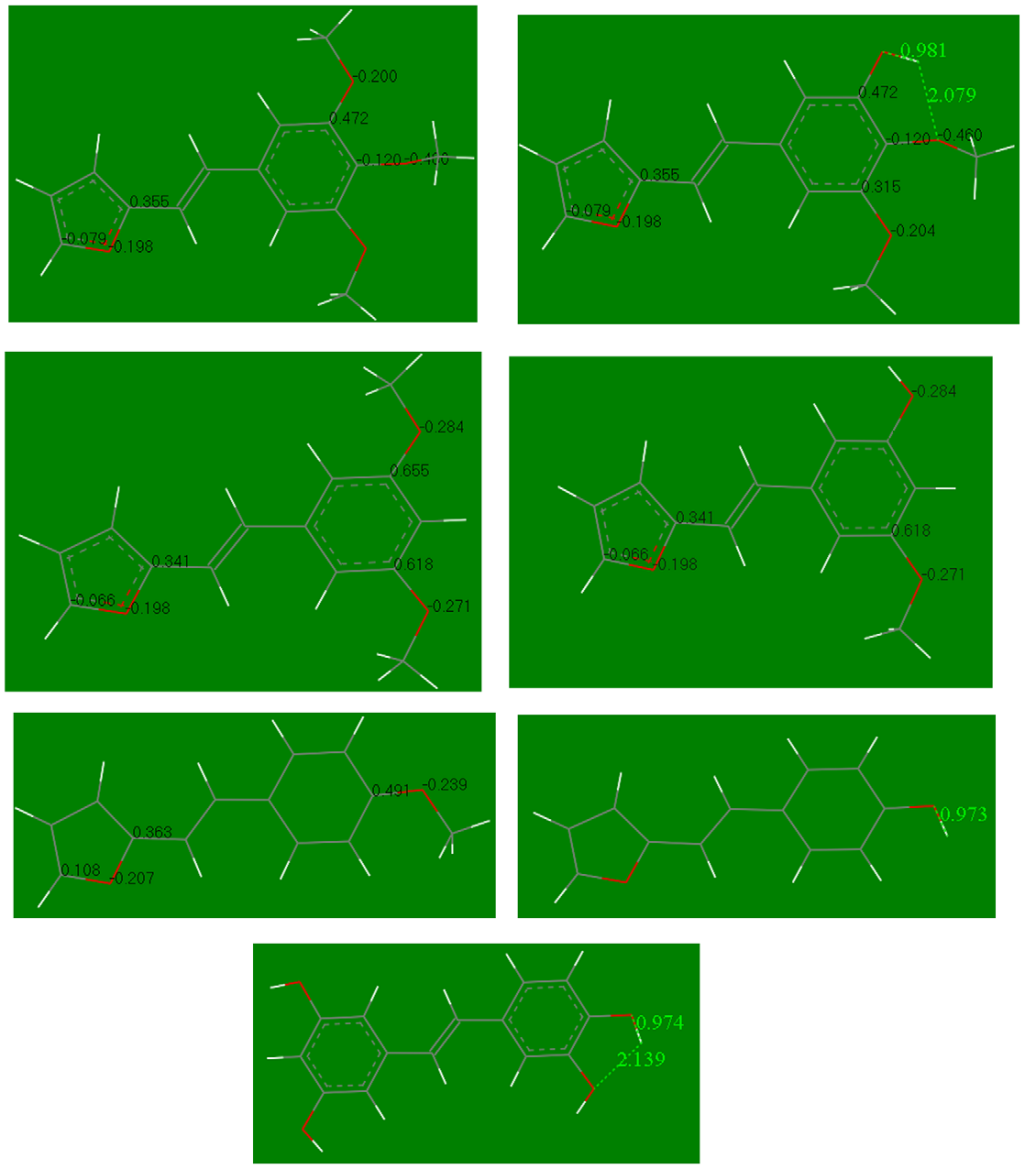
Selected population charge distribution. Compound **1**, top left, and its demethoxylated derivative (E)-4,5-dimethoxy,3-hydroxy-β-(2-furyl)-styrene, top right. Compound **3**, 2^nd^ group from top, left, and its demethoxylated derivative (E)-3-hydroxy-5-methoxy,β-(2-furyl)-styrene, 2^nd^ group from top, right. Compound **2**, 3^rd^ group from top, left, and its demethoxylated derivative (E)-4-hydroxy,β-(2-furyl)-styrene, 3^rd^ group from top, right. Geometry optimized molecular structure of piceatannol, bottom; some atomic separations are also included.

Since antifungal activity requires compounds **1**, **2** and **3** to cross the fungal membrane it was expected that compound **1** with its three methoxy groups would be the most effective; in contrast, the most active species was the dimethoxy compound **3**. However, under fungal attack, demethoxylation (or demethylation) of our compounds could produce hydroxyl formation. One mechanism to account for the lower activity of compound **1** is its incomplete demethoxylation and theoretical studies were performed to explore this hypothesis. Geometry optimizations of compounds **1**, **2** and **3**, and their population charges were calculated and analyzed (Figure 3). For the trimethoxy derivative **1** the highest positive charge (0.472) is located on position 3 of the aromatic ring (Figure 3A) suggesting this atom might be susceptible to a potential nucleophilic attack by a water molecule to generate an aromatic hydroxyl group [19]–[20].

Geometry optimization of the corresponding demethoxylated compound **1** derivative, (E)-4,5-dimethoxy,3-hydroxy-β-(2-furyl)-styrene, suggests further demethoxylation is more difficult (Figure 3A, right) since the atomic charge on the C-4 atom is much more negative (−0.120), whereas C-5 has a smaller charge (0.315) than a C adjacent to O(furyl) (0.335). In addition, this derivative shows a stabilizing interaction between H(hydroxyl) and the adjacent methoxy group: the H(hydroxyl) points to the O(methoxy) in position 4, with H-bond distance H - - O = 2.079 Å. Intramolecular hydrogen bonds are common and stabilizing features in polyhydroxy stilbenes; for instance, piceatannol shows a H-bond distance of interaction slightly longer, 2.139 Å (Figure 3D). Moreover, in (E)-4,5-dimethoxy,3-hydroxy-β-(2-furyl)-styrene the hydroxyl O-H bond is 0.981 Å (Figure 3A right), slightly longer than the equivalent hydroxyl bond length in piceatannol of 0.974 Å (Figure 3D). Repeating this analysis for compound **3**, with results shown in Figure 3B left, the C-3 atom of compound **3** has charge 0.655, and upon demethoxylation, the methoxy carbon at position C-5, retains its high charge (0.618) (Figure 3B right). Therefore, contrary to what happens to compound **1**, the first demethoxylation step in compound **3** does not affect the second demethoxylation step in its derivative (E)-3-hydroxy-5-methoxy,β-(2-furyl)-styrene. To complete these theoretical studies, we calculated charges on the monomethoxy compound **2**, and noted the C-ipso associated with C(methoxy) charge is 0.491 (Figure 3C left), and similar to that for the C(methoxy) in position 3 of compound **1** (0.472), (Figure 3A, left). In addition, compound **2**'s demethoxylated derivative, (E)-4-hydroxy,β-(2-furyl)-styrene has a shorter O-H bond distance, 0.973 Å (Figure 3C, right), than the O-H distance in (E)-4,5-dimethoxy,3-hydroxy-β-(2-furyl)-styrene, 0.981 Å (Figure 3A right), confirming that compound **1**'s hydroxyl group establishes an intramolecular H-bond interaction with the adjacent methoxy group, thereby making further demethoxylation of compound **1** difficult. These studies correlate with and suggest an explanation for the weaker antifungal activity of compound **1** compared to compound **3**.

Experimentally, we demonstrated that the antifungal activity of resveratrol is markedly lower than that of compounds **1**, **2** and **3**, and this may be related to the higher polarity that impedes diffusion through fungal membranes. Table 1 confirms this correlation: compound **3** has a Log*P* value of 4.3, closely related to the other methoxylated compounds, whereas resveratrol has a lower Log*P* (3.0). Also pterostilbene shows a higher log*P* than resveratrol, as expected, but not as high as compound **3**.

### Effect of the compound 3 on the production of laccases by *B. cinerea*

**Figure 4:**
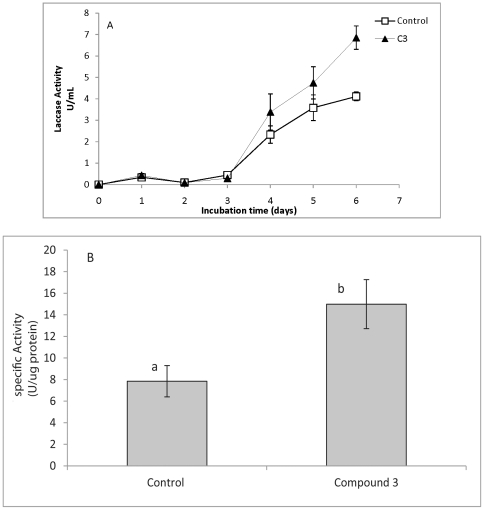
Effect of compound 3 on laccase production by *B. cinerea*. Kinetics of laccase activity in absence (□) or presence (▴) of 100 ug ml^−1^ of compound **3**, (**A**). Specific activity of laccase produced by *B. cinerea* after six days of incubation, (**B**). Different letters indicate that the means are significantly different at P<0.05.

The effect of compound **3** on the production of laccases by *B. Cinerea* was determined by inoculating pre-grown mycelium in minimum media containing compound **3** (Figure 4A); results showed increased production of laccases compared to the control group inoculated with just the solvent at the same concentration as treatment. Since the addition of compound **3** to the culture showed a difference in the mycelium growth, the enzymatic activity after six days of incubation was also expressed as U µg^−1^ of protein (Figure 4B). The enzymatic activity, in the presence of compound **3**, was twice that produced by the control.

### Effect of compound 3 on the cytoplasmatic membrane and on respiratory chain of *B. cinerea*

**Figure 5:**
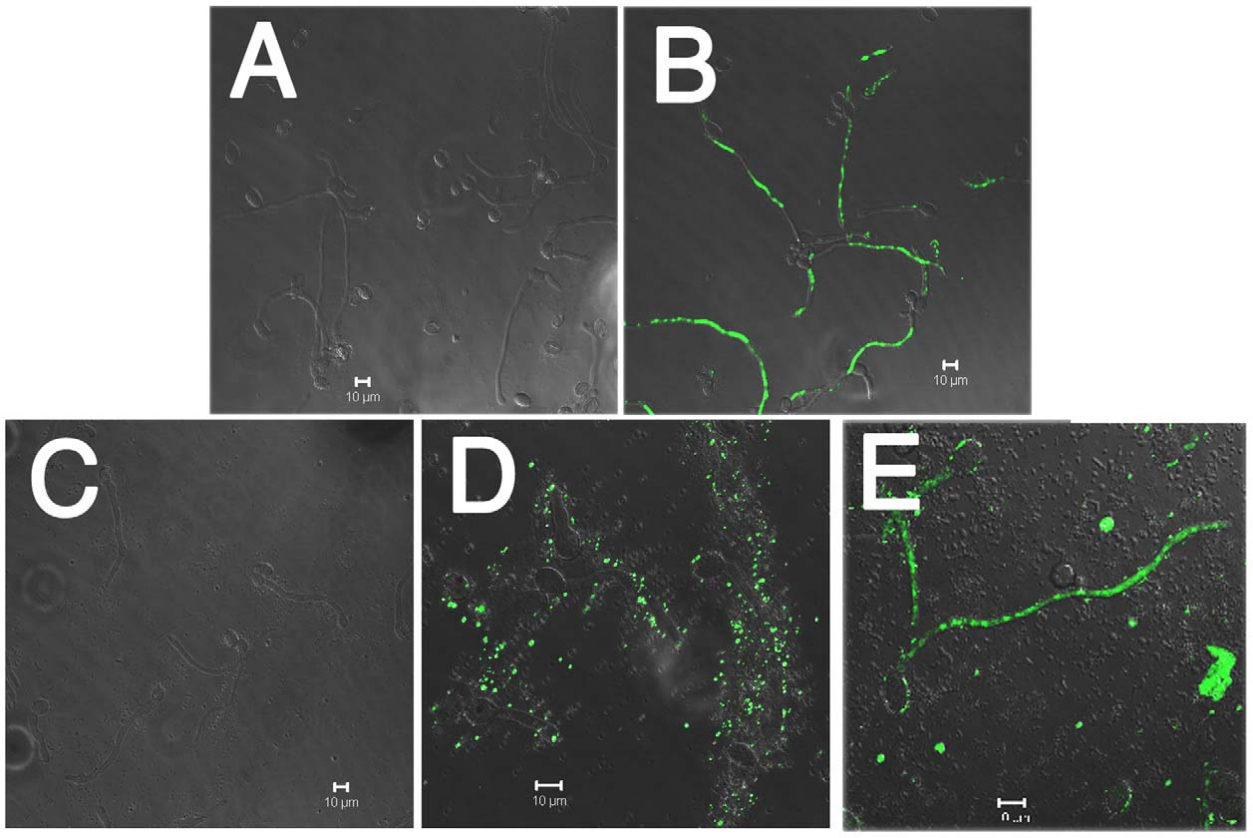
Effect of the compound 3 on the membrane integrity of *B. cinerea*. Conidia, at a final concentration of 1×10^5^ conidia ml^−1^, were incubated in liquid minimum medium at 22°C for 15 h in the presence of 8% (v/v) methanol-DMSO (A), 70% (v/v) ethanol (B) or 100 µg ml^−1^ of compound **3** for one (C), four (D) and six (E) hours.

Since reports that some stilbenes can interact with fungal membranes are known [10], the effect of compound **3** on the plasma membrane of *B. cinerea* was analyzed using Sytox Green staining (Figure 5). In the negative control, (methanol-DMSO), nuclei exhibit no fluorescence (Figure 5A), while when hyphae were treated with ethanol (positive control) fluorescent nuclei are observed, indicating alteration of the membrane integrity (Figure 5B). Treatment with compound **3** produced alteration of the *B. cinerea* plasma membrane after 6 h of incubation (Figures 5C–5E); after 4 h of incubation, an unspecific stain was observed, which did not correspond to nuclei fluorescence.

**Figure 6:**
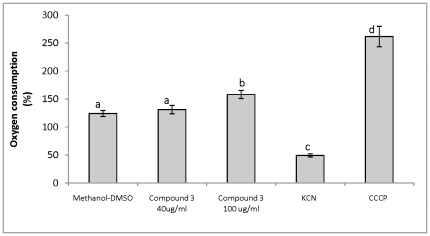
Effect of compound 3 on oxygen consumption by *B. cinerea* conidia.

In addition, resveratrol is known to inhibit the respiratory chain [8], [12] by affecting the rotary mechanism of F1-ATPase [21]. For this reason, the effect of compound **3** on the oxygen consumption of germinating conidia of this fungus was also analyzed (Figure 6). KCN, an inhibitor of the respiratory chain, and carbonyl cyanide *m*-chlorophenylhydrazone (CCCP), an uncoupler of the oxidative phosphorylation, were used as controls. In the presence of KCN, oxygen consumption decreased to 50%; KCN does not completely inhibit the oxygen consumption of *B. cinerea* conidia because this fungus contains a constitutive alternative oxidase [22]. The uncoupling compound CCCP increased oxygen consumption up to 250% while compound **3** at 40 µg ml^−1^ did not affect the oxygen consumption, although, at 100 µg ml^−1^ a slight increase of oxygen consumption was observed.

In this study, three 2-furyl derivatives of resveratrol with one, two and three methoxy groups were tested on *B. cinerea* isolate T50 and the results were compared to that obtained with resveratrol and the closely related dimethoxylated-resveratrol species, pterostilbene (3,5-dimethoxy-4′-hydroxy-trans-stilbene). Earlier studies show that pterostilbene is more active against *B. cinerea* than resveratrol on the mycelia growth of *B. Cinerea* [5]. Besides confirming these results, our study also demonstrates that compound **3** with two *meta* methoxy groups shows higher antifungal activity than mono- or tri-methoxylated furyl derivatives. The two dimethoxylated stilbene derivatives had different effects on *B. cinerea*: compound **3** was more active on mycelial growth, whereas pterostilbene was a more effective inhibitor of conidia germination.

Therefore, the presence of two *meta* methoxy groups in stilbene seems to be an important factor for antifungal activity against *B. cinerea* while the presence of furyl or phenolic groups determines the target of action of these compounds.

The high antifungal activity of compound **3** against *B. cinerea* can be explained through a fungal-mediated demethoxylation reaction to generate the aromatic hydroxyl groups that are considered to be important structural features in stilbenes for free radical scavenging and prooxidant activity [23]. Our theoretical DFT calculations seem to support the production of these reactive hydroxyl groups based on the higher antifungal activity of compound **3**. Another potential mechanism based on quinone formation by laccases, upon hydroxyl formation, may be also expected.

Hydrophobicity is also an important factor for effective fungicidal activity that can be evaluated through Log*P* values. Earlier studies found an apparently optimum hydrophobic effect for antifungal activity as molecules with high and low Log*P* value were less active [24]–[26]. In our study, molecules with a Log*P* value slightly higher than 4.0 showed higher antifungal activities against *B. cinerea*, compared to resveratrol (log*P* of 3.0). Table 1 confirms that the presence of two methoxy groups increases the hydrophobicity of the compounds, compared to resveratrol, which suggests increased possibility of crossing, or at least interaction with, the fungal cytoplasmic membrane.

Earlier studies to determine the target of pterostilbene on *B. cinerea* suggested that the endoplasmic reticulum and mitochondrial membranes were affected [10]. Our studies with compound **3** show alteration of the plasma membrane after 6 h of incubation. Recently, it was shown that, in *Saccharomyces cerevisiae*, pterostilbene increased the gene expression involved in mitochondrial functions, drug detoxification and lipid metabolism significantly [27]. Our results show that, at low concentration, compound **3** does not affect the respiratory chain of *B. cinerea* conidia while at high concentration it could act as an uncoupler.

That methoxylated stilbenes can react by other mechanisms, such as inhibition of cytochrome P450 enzymes or apoptosis induction, cannot be discarded. Pterostilbene inhibits human recombinant cytochrome P450, CYP1A1 and CYP1B1 [16], while its corresponding demethoxylated stilbene (resveratrol) does not. It was also shown that pterostilbene and 3′-hydroxypterostilbene, the natural 3,5-dimethoxy analog of piceatannol, but not resveratrol and piceatannol, induce apoptosis in tumor cells [18] and that both compounds, were able to induce apoptosis, in the two Fas-ligand resistant lymphoma cell lines, HUT78B1 and HUT78B3, and the multi drug-resistant leukemia cell lines HL60-R and K562-ADR (a Bcr-Abl-expressing cell line resistant to imatinib mesylate) [17]. We conclude that compounds **1**, **2** and **3** could have additional relevant biological properties, as recently indicated for A549 lung carcinoma [28], and inhibition of lipopolysaccharide-induced nitric oxide production [29], where, in contrast with our results, the trimethoxy derivative **1** was the most active.

Phenolic compounds such as resveratrol induce laccase production in *B. cinerea*, and these enzymes participate in the oxidation of these compounds which aids in their biological activity [6]. Our studies demonstrate that non-phenolic compounds as compound **3** also increase laccase production.

In conclusion, antifungal activity and target site on *B. cinerea* can be modulated through structural modifications of compounds. The two principal features that differ among the title compounds **1**, **2** and **3** and resveratrol (and pterostilbene) are: increasing number of methoxy groups and the 2-furyl moiety. The presence of methoxy groups improves the antifungal activity of stilbene derivatives while the presence of furyl or phenyl groups determines the biological target on the fungus. Methoxylation is an important molecular feature for membrane penetration while subsequent demethoxylation may be necessary to make hydroxyls available for increasing antifungal activity. This latter process seems to be more efficient with the dimethoxy species **3** than the trimethoxy derivative **1**. The specific role of the 5-membered ring will be investigated synthesizing structurally modified 2-furyl derivatives that will be accompanied by antifungal tests.

## Materials and Methods

### Ethics Statement

An ethics statement is not required for this work.

### Test compounds

The compounds used in this study (Figure 1) were the natural compounds resveratrol and pterostilbene, (Sigma Chemical Co., St. Louis, MO, USA), and synthetic (E)-3,4,5-trimethoxy-β- (2-furyl)-styrene (**1**), (E)-4-methoxy-β-(2-furyl)-styrene (**2**) and (E)-3,5-dimethoxy-β-(2-furyl)- styrene (**3**). The synthesis of compounds **1**, **2** and **3**, based on the Wittig reaction [30]–[31], differs from that reported in the literature. NMR experiments were performed using a Varian XL-300 spectrometer, IR spectra were taken with a Shimadzu IR-740 instrument and MS spectra were recorded by an HP5971A/MS detector coupled with HP5890 gas chromatograph.

#### Synthesis of (E)-4-Trimethoxy-β-(2-furyl)-styrene, compound 1

NaBH_4_ (18 mmol) was added to a solution of 1,2,3-trimethoxy-5-benzaldehyde (18 mmol) in MeOH (18 ml) at 0°C. After 30 min the reaction mixture was concentrated under reduced pressure. The raw material was dissolved in ethyl ether (100 ml) and washed with a saturated solution of NaCl. The organic layer was dried with anhydrous Na_2_SO_4_ and the ether eliminated *in vacuo*. The obtained alcohol derivative, 1,2,3-trimethoxy-5-benzyl alcohol, (16.6 mmol) was dissolved in anhydrous CH_2_Cl_2_ and stirred at 0°C, then PBr_3_ (8.3 mmol) was added and the temperature raised to 25°C; after 30 min the alcohol was completely reacted as seen from TLC (n-hexane/ethyl acetate). Water was then added and ethyl ether (3×30 ml) used for extraction. The organic phase was washed with a saturated solution of NaCl and dried with anhydrous Na_2_SO_4_.

Upon ether elimination at low pressure, 15 mmol of the corresponding benzyl bromide were obtained. This was dissolved in toluene (150 ml) and 15 mmol di Ph_3_P were added with stirring. After refluxing for 12 h and TLC checking (n-hexane/ethyl acetate 9∶1), the obtained suspension was decanted at room temperature and filtered. The product was washed with cold toluene and 13.9 mmol of the phosphonium salt were obtained. This was dissolved in 100 ml of isopropyl alcohol and 28.2 mmol of LiOH·H_2_O were added. This solution was stirred and refluxed for 15 min. 2-Formyl-furane (13.9 mmol) was then added and refluxing performed until disappearance of the furyl aldehyde (TLC, n-hexane/ethyl acetate 9∶1), achieved in 3 h. The solution was brought to 25°C and the solvent eliminated at low pressure. The product was dissolved in CHCl_3_ (10 ml) and the resulting solution introduced in a silica gel column (h = 10 cm, Φ = 4 cm) using n-hexane/ethyl acetate 10∶1 as eluent. The obtained olefin (13.2 mmol) is a mixture of both diastereoisomers E/Z ca. 1∶1. The *cis-trans* isomerization was performed by dissolving in CHCl_3_ and adding iodine (1.3 mmol) in 12 h, as followed with TLC. The organic solution was separated and treated first with a saturated solution of sodium thiosulphate and later with a saturated solution of NaCl. After drying with anhydrous Na_2_SO_4_, 11.8 mmol of the expected (E)-3,4,5- trimethoxy-β-(2-furyl)-styrene were obtained. Compounds **2** and **3** were synthesized in analogous manner, but using 1-methoxy-4-benzaldehyde (for compound **2**) and 1,3-dimethoxy-5-benzaldehyde (for compound **3**), respectively, as starting material, instead of 1,2,3-trimethoxy-5-benzaldehyde for compound **1**. The scheme synthesis of compound **1** is shown Figure S1, those of compounds **2** and **3** are in Figure S2.

### Spectroscopic data

#### (E)-3,4,5-Trimethoxy-β-(2-furyl)-styrene (compound 1)

**^1^H-NMR**(CDCl_3_),δ(ppm): 3.86 (s, 3H, OC**H_3_**); 3.91 (s, 6H, OC**H_3_**); 6.36 (d, 1H, J = 2.93 Hz, **H_2_**-furan); 6.45 (dd 1H J_1_ = J_2_ = 2.93 Hz, **H_3_**-furan); 6.70 (s, 2H, **H_2_-H_6_**-Ar); 6.79 (d, 1H, J = 16.8 Hz, **H**olefin); 6.97 (d, 1H, J = 16.8 Hz, **H**-olefin); 7.39 (bd, 1H, J = 2.93 Hz, **H_4_**-furan). **^13^C-NMR**(CDCl_3_), δ(ppm): 55.86 (m-**C**H_3_); 60.65 (p-**C**H_3_); 103.35 (**C_2_-C_6_**-Ar); 108.16 (**C_2_**-furan):111.45(**C_3_**-furan); 115.85 (**C_β_**-olefin); 126.9 (**C_1_**-Ar) 132.5 (**C_α_**-olefin); 137.88 (**C_4_**-Ar); 141.85 (**C_1_**-furan); 152.96 (**C_4_**-furan); 153.20 (**C_3_-C_5_**-Ar). **IR** (CHCl_3_, 1%), ν(cm^−1^): 1598; 1470; 1459; 1253; 1154. **MS** m/z: 260 (M_+_) [29], [32]–[33]. MP: 78.7–79.8°C. Yield, **75%** (3.07 g), colorless.

#### (E)-4-Methoxy-β-(2-furyl)-styrene (compound 2)

**^1^H-NMR**(CDCl_3_), δ(ppm): 3.82 (s, 3H, OC**H_3_**); 6.29 (d, 1H, J = 2.93 Hz, **H_2_**-furan); 6.40 (d, 1H, J = 2.93 Hz, **H_3_**-furan); 6.75 (d, 1H, J = 16.8 Hz, **H**-olefin); 6.87 (bd, 2H, J = 8.8 Hz, **H_3_-H_5_**-Ar); 6.98 (d, 1H, J = 16.8 Hz, **H**-olefin); 7.39 (bd, 2H, J = 8.8 Hz, **H_2_-H_5_**-Ar). 7.40 (d, 1H, J = 2.93 Hz, **H_4_**-furan). **^13^CNMR**(CDCl_3_), δ(ppm): 55.16 (**C**H_3_); 107.57 (**C_2_**-furan); 111.48 (**C_3_**-furan); 114.15 (**C_3_**-**C_5_**, Ar); 126.84 (**C_β_**-olefin); 127.5 (**C_2_**-**C_6_**-Ar); 128.12 (**C_1_**-Ar); 129.83 (**C_α_**-olefin); 141.67 (**C_1_**-furan); 153.6 (**C_4_**-furan); 159.33 (**C_4_**-Ar). **IR** (CHCl_3_, 1%), ν(cm^−1^): 1642; 1560. **MS** m/z: 200 (M_+_) [34]–[40]. MP: 73.7–74.3°C [34], [37]. Yield, **72%** (2.64 g), colorless.

#### (E)-3,5-Dimethoxy-β-(2-furyl)-styrene (compound 3)

**^1^HNMR**(CDCl_3_), δ(ppm): 3.82 (s, 6H, OC**H_3_**); 6.37 (d, 1H, J = 2.93 Hz, **H_2_**-furan); 6.38 (d, 1H,J = 2.19 Hz, **H_4_**-Ar); 6.42 (dd 1H J_1_ = J_2_ = 2.93 Hz, **H_3_**-furan); 6.61 (d, 2H, J = 2.19 Hz, **H_2_-H_6_**-Ar); 6.84 (d, 1H, J = 16.8 Hz, **H**-olefin); 6.95 (d, 1H, J = 16.8 Hz, **H**-olefin); 7.40 (bd, 1H, J = 2.93 Hz, **H_4_**-furan) **^13^C-NMR**(CDCl_3_), δ(ppm): 54.8 (**C**H_3_); 99.8 (**C_4_**-Ar). 104.1 (**C_2_**-**C_6_**-Ar); 110.1 (**C_2_**-furan); 111.4 (**C_3_**-furan); 116.7 (**C_α_**-olefin); 126.9 (**C_β_**-olefin); 138.9(**C_1_**-Ar). 141.9 (**C_1_**-furan); 153.6 (**C_4_**-furan); 160.8 (**C_3_**-**C_5_**-Ar). **IR** (CHCl_3_, 1%), ν(cm^−1^): 1602; 1470; 1430; 1200; 1150. **MS** m/z: 230 (M_+_) [29], [32], [38], [41]–[43]. MP: 105.9–107.7°C. Yield, **70%** (2.88 g), colorless.

The H-NMR of compounds **1**, **2** and **3**, are depicted in Figures S3, S4 and S5. The C-NMR of compound **2** is shown in Figure S6 and that of compound **3** in Figure S7.

### Fungal isolate and culture condition

In this study, isolate T50 of *B. cinerea* was used. This was originally isolated from a naturally infected tomato (*Solanum lycopersicum* cv. Roma) [44] and was maintained on malt-yeast extract agar slants [2% (w/v) malt extract, 0.2% (w/v) yeast extract and 1.5% (w/v) agar] at 4°C. The fungus was grown in the dark on malt-yeast extract agar medium [2% (w/v) malt extract, 0.2% (w/v) yeast extract and 1.5% (w/v) agar) or soft agar (2% (w/v) malt extract, 0.2% (w/v) yeast extract and 0.6% (w/v) agar]. In the mechanism of action analysis, liquid minimum medium [KH_2_PO_4_ (1 g l^−1^), K_2_HPO_4_ (0.5 g l^−1^), MgSO_4_·7H_2_O (0.5 g l^−1^), KCl (0.5 g l^−1^), FeSO_4_·7H_2_O (0.01 g l^−1^)] pH 6.5, 25 mol l^−1^ ammonium tartrate as a nitrogen source, and 1% (w/v) glucose as carbon source were used.

### Effect of the compounds on the mycelial growth of *B. cinerea* on solid media

The fungitoxicity of the compounds was assessed using the radial growth test on malt-yeast extract agar [45]. Test compounds were dissolved in methanol-DMSO (24∶1) and added at final concentrations of 10, 20, 40, 80 or 100 µg ml^−1^. The final methanol-DMSO concentration was identical in control and treatment assays. Mycelial growth diameters were measured daily. After 96 hours of incubation the inhibition percentages relative to the control with methanol-DMSO were calculated. Results were expressed as effective concentration (ED_50_) that reduced mycelial growth by 50%, determined by regressing the inhibition of radial growth values (percent control) against the values of compound concentration. These experiments were done in triplicate.

### Effect of the compounds on germination of *B. cinerea* conidia

Conidial germination assays were carried out on microscope slides coated with soft agar medium (2 mm thickness). The added compounds were dissolved in methanol-DMSO (24∶1) at a final concentration of 10 or 100 µg ml^−1^. The slides were inoculated with dry conidia obtained from sporulated mycelia (1 week old), placed in a humid chamber (90% relative humidity), and incubated in the dark at 22°C for 7 h. Control contained methanol-DMSO at the same concentration as treatments. Conidial germination was determined directly on the slides at intervals of 1 h. The percentage of germination was estimated by counting the number of germinated conidia in five microscope fields each containing approximately 40 conidia. Conidia were considered germinated when the germ tube length was equal to or greater than conidial diameter. These experiments were done in triplicate.

### Effect of compound 3 on the production of laccases by *B. cinerea*

50 ml Erlenmeyer flasks containing 5 ml of minimum medium in the presence of 1% (w/v) of glucose were inoculated with conidia (1×10^6^ conidia ml^−1^) and incubated for three days at 22°C in static conditions. After this incubation time, culture media were discarded and fresh minimum medium without glucose containing of 100 µg ml^−1^ of compound **3** or methanol-DMSO, at the same concentration as treatments, was added to mycelium. Cultures were incubated at 22°C and laccase activity was determined in the supernatants. To evaluate laccase activity, syringaldazine was used as the substrate [46]. Enzyme activity was determined spectrophotometrically by monitoring the absorbance at 530 nm. The reaction mixture (1 ml) contained 30 µl of culture filtrates and 0.1 mmol l^−1^ syringaldazine in 50 mmol l^−1^ sodium phosphate buffer (pH 6.0) and was incubated at 22°C for 15 min. One unit per ml of laccase (U ml^−1^) corresponds to the amount of enzyme that increases the absorbance in 0.1 in 1 min. Protein concentration was determined as described [47].

### Determination of the effect of compound 3 on the membrane integrity of *B. cinerea*

This was determined using the SYTOX Green uptake assay [48]. *B. cinerea* conidia at a final concentration of 1×10^5^ conidia ml^−1^ were inoculated in 24-well plates (lined with 12-mm glass coverslips) containing 1 ml of liquid minimum medium. Cultures were incubated at 22°C for 15 h to permit the germination of the conidia. After this time, liquid medium was removed and same medium with 70% (v/v) ethanol (positive control), 8% (v/v) methanol-DMSO (negative control), or 20, 40 and 100 µg ml^−1^ of compound **3** was added to each well. The mixtures were incubated at 22°C for one, four and six hours in the case of compound **3** and methanol-DMSO or for 10 min when ethanol was used. *B. cinerea* hyphae adhered to glass coverslips were washed three times with liquid minimum medium and were stained with 50 nmol l^−1^ SYTOX Green (Molecular Probes, Eugene, OR, USA). After 10 min of incubation, the hyphae were washed with minimum medium and glass coverslips containing hyphae were mounted in slides. For the assembly of the samples in the slides, 15 µl of DABCO (1,4-diazabicyclo[2.2.2]octane) was used. The fluorescence of *B. cinerea* hyphae stained with SYTOX Green was observed under a confocal microscope (Carl Zeiss LSM 510) at an excitation wavelength of 488 nm and an emission wavelength of 540 nm. These experiments were done at least in triplicate.

### Determination of the effect of compound 3 on the oxygen consumption of *B. cinerea* conidia

Oxygen consumption was determined polarographically at 25°C with a Hansatech oxygen electrode by using germinating conidia in a total volume of 1 ml. To obtain conidia in suspension, Murashige and Skoog's basal medium at 4.4 g l^−1^ (Phytotechnology Laboratories, Lenexa, KS, USA) was added to Petri dishes containing conidia. The conidia were harvested by scraping with a sterile spatula. To eliminate mycelium, the suspension was filtered through glass wool. The conidia concentration was adjusted to 1×10**^7^** conidia ml^−1^ with minimum liquid media, in the presence of 2% (w/v) glucose. Conidia were incubated for 2 hours at 22°C. The measurement of basal oxygen consumption was carried out for 2 min in the same minimum liquid medium. After this time, 0.05 M carbonyl cyanide *m*-chlorophenylhydrazone (CCCP), 10 mmol l^−1^ KCN or the compound **3**, dissolved in methanol-DMSO (24∶1) at a final concentration of 40 or 100 µg ml^−1^ were added. Control contained methanol-DMSO at the same concentration as treatments. Oxygen consumption was determined for eight more minutes. These experiments were done at least in triplicate.

### Statistical analyses

Data presented in Figures 2, 4 and 6 are expressed as mean ± SD from at least three independent results. Significant differences were determined using a one way analysis of variance (Microsoft Office Excel 2007). Mean values were separated using the least significant difference test (*P*<0.05).

### Theoretical calculations

Lipophilicity values were taken from SciFinder database, originally calculated using Advanced Chemistry Development (ACD/Labs) Software V9.04 (1994–2010 ACD/Labs). Geometry optimization for the 3-furyl resveratrol derivatives was performed with theoretical methods using the density functional theory (DFT) program DMol3, implemented in Materials Studio 4.4 (PC platform) from Accelrys (San Diego, USA). The density setting was the general gradient approximation (GGA) [49] and the Becke exchange (BP) functional [50]. A double numeric basis set with polarization functions (DNP) for an all electron calculation was used [51]. The same conditions were employed to calculate frequencies, useful to determine geometries in a minimum of energy, and population charges. In this *ab-initio* study the approximate co-planarity of the molecules was assumed because resveratrol and its derivatives prefer this resonance stabilized conformation. In this study we do not investigate the mechanism of hydrolysis, which may be demethoxylation [19]–[20] or demethylation. Therefore, by using the word demethoxylation we mean hydroxyl formation, and do not exclude the demethylation mechanism.

### Procedure information about figures

#### Figure 2

Compounds were dissolved in methanol-DMSO (24∶1). The slides were inoculated with dry conidia, placed in a humid chamber, and incubated at 22°C. Percentages of germination relative to control were determined after 7 h of incubation. Each bar represents the mean of at least three independent experiments ± standard deviation.

#### Figure 4

Each bar represents the mean of at three independent experiments ± standard deviation. The final methanol-DMSO concentration was identical in control and treatment assays.

#### Figure 5

The fluorescence of *B. cinerea* hyphae stained with SYTOX Green was observed under a confocal microscope. These photographs are representatives of five independent experiments.

#### Figure 6

1×10^7^ Conidia ml^−1^ were suspended in minimum liquid media, in the presence of 2% (w/v) glucose. Oxygen concentration was determined in the presence of methanol-DMSO at the same concentration as treatments, compound **3** at 40 or 100 µg ml^−1^, 10 mM KCN or 0.05 M carbonyl cyanide *m*-chlorophenylhydrazone (CCCP). Percentage of oxygen consumption was determined relative to control in the absence of methanol-DMSO.

## Supporting Information

Figure S1 **Scheme synthesis of compound 1.**
(TIF)

Figure S2 **Scheme synthesis of compound 2 and 3.**
(TIF)

Figure S3 **H-NMR of compound 1.**
(TIF)

Figure S4 **H-NMR of compound 2.**
(TIF)

Figure S5 **H-NMR of compound 3.**
(TIF)

Figure S6 **C-NMR of compound 2.**
(TIF)

Figure S7 **C-NMR of compound 3.**
(TIF)

## References

[1] Elad Y, Evensen K (1995). Physiological aspects of resistance to *Botrytis cinerea*.. Phytopathology.

[2] Latorre BA, Flores V, Sara AM, Roco A (1994). Dicarboximide-resistant strains of *Botrytis cinerea* from table grapes in Chile: survey and characterization.. Plant Dis.

[3] Grayer RJ, Kokubun T (2001). Plant-fungal interactions: the search for phytoalexins and other antifungal compounds from higher plants.. Phytochemistry.

[4] Chong J, Poutaraud A, Hugueney P (2009). Metabolism and roles of stilbenes in plants.. Plant Sci.

[5] Adrian M, Jeandet P, Veneau J, Weston L, Besis R (1997). Biological activity of resveratrol, a stilbenic compound from grapevines, against *Botrytis cinerea*, the causal agent for gray mold.. J Chem Ecol.

[6] Schouten A, Wagemakers L, Stefanato FL, van der Kaaij RM, van Kan JAL (2002). Resveratrol acts as a natural profungicide and induces self-intoxication by a specific laccase.. Mol Microbiol.

[7] Baldrian P (2006). Fungal laccases – occurrence and properties.. Fems Microbiol Rev.

[8] Hart JH (1981). Role of phytostilbenes in decay and disease resistant.. Ann Rev Phytopathol.

[9] Pezet R, Pond V, Daniel M, Purkayastha RP (1995). Mode of action of Vitaceae stilbenes on fungal cells.. “Handbook of phytoalexin metabolism and action”.

[10] Pezet R, Pond V (1990). Ultrastructural observations of pterostilbene fungitoxicity in dormant conidia of *Botrytis cinerea*.. Pers J Phytopathol.

[11] van Baarlen P, Legendre L, van Kan JAL, Elad Y, Williamson B, Tudzynski P, Delen N (2004). Plant defense compounds against *Botrytis* infection.. “Botrytis: Biology, pathology and control”.

[12] Sbaghi M, Jeandet P, Bessis R, Leroux P (1996). Degradation of stilbene-type phytoalexins in relation to the pathogenicity of *Botrytis cinerea* to grapevines.. Plant Pathol.

[13] Pezet R, Pont V, Hoang-Van K (1991). Evidence for oxidative detoxication of pterostilbene and resveratrol by a laccase-like stilbene oxidase produced by *Botrytis cinerea*.. Physiol Mol Plant Pathol.

[14] Rossi M, Caruso F, Opazo C, Salciccioli J (2008). Crystal and molecular structure of piceatannol; Scavenging features of resveratrol and piceatannol on hydroxyl and peroxyl radicals and docking with transthyretin.. J Agric Food Chem.

[15] Caruso F, Tanski J, Villegas-Estrada A, Rossi M (2004). Structural basis for antioxidant activity of trans-resveratrol: ab initio calculations and crystal and molecular structure.. J Agric Food Chem.

[16] Mikstacka R, Przybylska D, Rimando AM, Baer-Dubowska W (2007). Inhibition of human recombinant cytochromes P450 CYP1A1 and CYP1B1 by trans-resveratrol methyl ethers.. Mol Nutr Food Res.

[17] Tolomeo M, Grimaudo S, Di Cristina A, Roberti M, Pizzirani D (2005). Pterostilbene and 3′-hydroxypterostilbene are effective apoptosis-inducing agents in MDR and BCR-ABL-expressing leukemia cells.. Int J Biochem Cell Biol.

[18] Roberti M, Pizzirani D, Simoni D, Rondanin R, Baruchello R (2003). Synthesis and biological evaluation of resveratrol and analogues as apoptosis-inducing agents.. J Med Chem.

[19] Meunier G, Meunier B (1985). Evidences for an efficient demethylation of methoxyellipticine derivatives catalyzed by a peroxidise.. J Amer Chem Soc.

[20] Sobarzo-Sanchez E, Castedo L, De la Fuente JR (2006). Synthesis and theoretical study on 5,6-dimethoxy-2,3-dihydro-7H-dibenzo[de,h]quinolin-7-one: Possible precursor on the aromatic demethoxylation in oxoisoaporphines.. Struct Chem.

[21] Gledhill JR, Montgomery MG, Leslie AGW, Walker JE (2007). Mechanism of inhibition of bovine F_1_-ATPase by resveratrol and related polyphenols.. Proc Natl Acad Sci USA.

[22] Tamura H, Mizutani A, Yukioka H, Miki N, Ohba H (1999). Effect of the methoxyiminoacetamide fungicide, SSF129, on respiratory activity in *Botrytis cinerea*.. Pestic Sci.

[23] Murias M, Jäger W, Handler N, Erker T, Horvath Z (2005). Antioxidant, prooxidant and cytotoxic activity of hydroxylated resveratrol analogues: structure-activity relationship.. Biochem Pharmacol.

[24] Yang G, Jiang X, Yang H (2002). Development of novel pesticides based on phytoalexins: Part 2. Quantitative structure-activity relationships of 2-heteroaryl-4-chromanone derivatives.. Pest Manag Sci.

[25] Voda K, Boh B, Vrtacnik M (2004). A quantitative structure–antifungal activity relationship study of oxygenated aromatic essential oil compounds using data structuring and PLS regression analysis.. J Mol Model.

[26] Niewiadomy A, Matysiak J, Fekner Z, Czeczco R (2006). Synthesis, antifungal activity and SAR of *N*-substituted and *N*, *N*-disubstituted 2,4-dihydroxythiobenzamides.. J Pestic Sci.

[27] Pan Z, Agarwal AK, Xu T, Feng Q, Baerson SR (2008). Identification of molecular pathways affected by pterostilbene, a natural dimethylether analog of resveratrol.. BMC Med Gen.

[28] Kim S, Min SY, Lee SK, Cho W-J (2003). Comparative Molecular Field Analysis Study of Stilbene Derivatives Active against A549 Lung Carcinoma.. Chem Pharm Bull.

[29] Lee SK, Min HY, Huh SK, Kim EY, Lee EJ (2003). Styrylheterocycles: a novel class of inhibitors on lipopolysaccharide-induced nitric oxide production.. Bioorg Med Chem Lett.

[30] Tofi M, Georgiou T, Montagnon T, Vassilikogiannakis G (2005). Regioselective ortho lithiation of 3-aryl and 3-styryl furans.. Org Lett.

[31] Antonioletti R, Bonadies F, Ciammaichella A, Viglianti A (2008). Lithium hydroxide as base in the Wittig reaction. A simple method for olefin synthesis.. Tetrahedron.

[32] Kim S-H, Chun Y-J (2003). Stilbene derivative with cytochrome P450 1B1 inhibitory activity, pharmaceutically acceptable salt, preparation method, pharmaceutical composition, and use in prevention of carcinogenesis.. PCT Int Appl.

[33] Kim S, Min SY, Lee SK, Cho W-J (2003). Comparative molecular field analysis study of stilbene derivatives active against A549 lung carcinoma.. Chem Pharm Bul.

[34] Wu J-Y, Ho J-H, Shih S-M, Hsieh T-L, Ho T-I (1999). Solvent-Dependent Photochemical Rearrangements of Ethers of Styrylheterocycles.. Org Lett (1999).

[35] Aun CE, Clarkson TJ, Happer DAR (1990). β-Substituent effects in the carbon-13 NMR chemical shifts of styrenes.. J Chem Soc Perkin Trans.

[36] Wang Z, Ding Q, He X, Wu J (2009). Palladium-catalyzed decarboxylative cross-coupling reaction of cinnamic acid with aryl iodide.. Org Biomol Chem.

[37] Chu L-T, Yu P-C, Wu B-J, Liao Y-C, Chou C-H (2007). Synthesis of areno[e]indenes by the flash vacuum pyrolysis of 4-methoxystyryl arenes.. Heterocycles.

[38] Lee SK, Park E-J, Lee E, Min H-Y, Kim E-Y (2004). Styrylheterocycles as a novel class inhibitor of cyclooxygenase-2-mediated prostaglandin E2 production.. Bioorg Med Chem Lett.

[39] Ho J-H, Ho T-I, Liu RSH (2001). Proton-Assisted Switching of Reaction Pathways of Stilbene Analogs Brought by Direct Irradiation.. Org Lett.

[40] Ho T-I, Ho J-H, Wu J-Y (2000). Novel Acid-Catalyzed Hydrolysis of an Intermediate from a Photorearrangement of Stilbenes.. J Amer Chem Soc.

[41] Meng X-L, Yang J-Y, Chen G-L, Wang L-H, Zhang L-J (2008). Effects of resveratrol and its derivatives on lipopolysaccharide-induced microglial activation and their structure-activity relationships.. Chem-Biol Interact.

[42] Katsuyama Y, Funa N, Horinouchi S (2007). Precursor-directed biosynthesis of stilbene methyl ethers in Escherichia coli.. Biotech J.

[43] Kim S, Ko H, Park JE, Jung S, Lee SK (2002). Design, Synthesis, and Discovery of Novel trans-Stilbene Analogues as Potent Selective Human Cytochrome P450 1B1 Inhibitors.. J Med Chem.

[44] Muñoz G, Hinrichsen P, Brygoo Y, Giraud T (2002). Genetic characterisation of *Botrytis cinerea* populations in Chile.. Mycol Res.

[45] Mendoza L, Araya-Maturana R, Cardona W, Delgado-Castro T, García C (2005). In vitro sensitivity of *Botrytis cinerea* to anthraquinone and anthrahydroquinone derivates.. J Agric Food Chem.

[46] Chefetz B, Chen Y, Hadar Y (1998). Purification and characterization of laccase from *Chaetomium thermophilium* and its role in humification.. Appl Env Microbiol.

[47] Bradford M (1976). A rapid and sensitive method for the quantitation of microgram quantities of protein utilizing the principle of protein-dye binding.. Anal Biochem.

[48] Thevissen K, Terras FRG, Broekaert WF (1999). Permeabilization of fungal membranes by plant defensins inhibit fungal growth.. Appl Environ Microbiol.

[49] Perdew JP, Chevary JA, Vosko SH, Jackson KA, Pederson MR (1992). Atoms, molecules, solids, and surfaces: applications of the generalized gradient approximation for exchange and correlation.. Phys Rev B: Cond Mat Mater Phys.

[50] Delley B (2000). From molecules to solids with the DMol3 approach.. J Chem Phys.

[51] Delley B (1996). Fast calculation of electrostatics in crystals and large molecules.. J Phys Chem.

