# Supplementary figures and images for: Antifungal Activity of Resveratrol against Botrytis cinerea Is Improved Using 2-Furyl Derivatives
Source: PLoS One. 2011 Oct 11;6(10):e25421. doi: 10.1371/journal.pone.0025421 (PMC3191159; doi:10.1371/journal.pone.0025421)

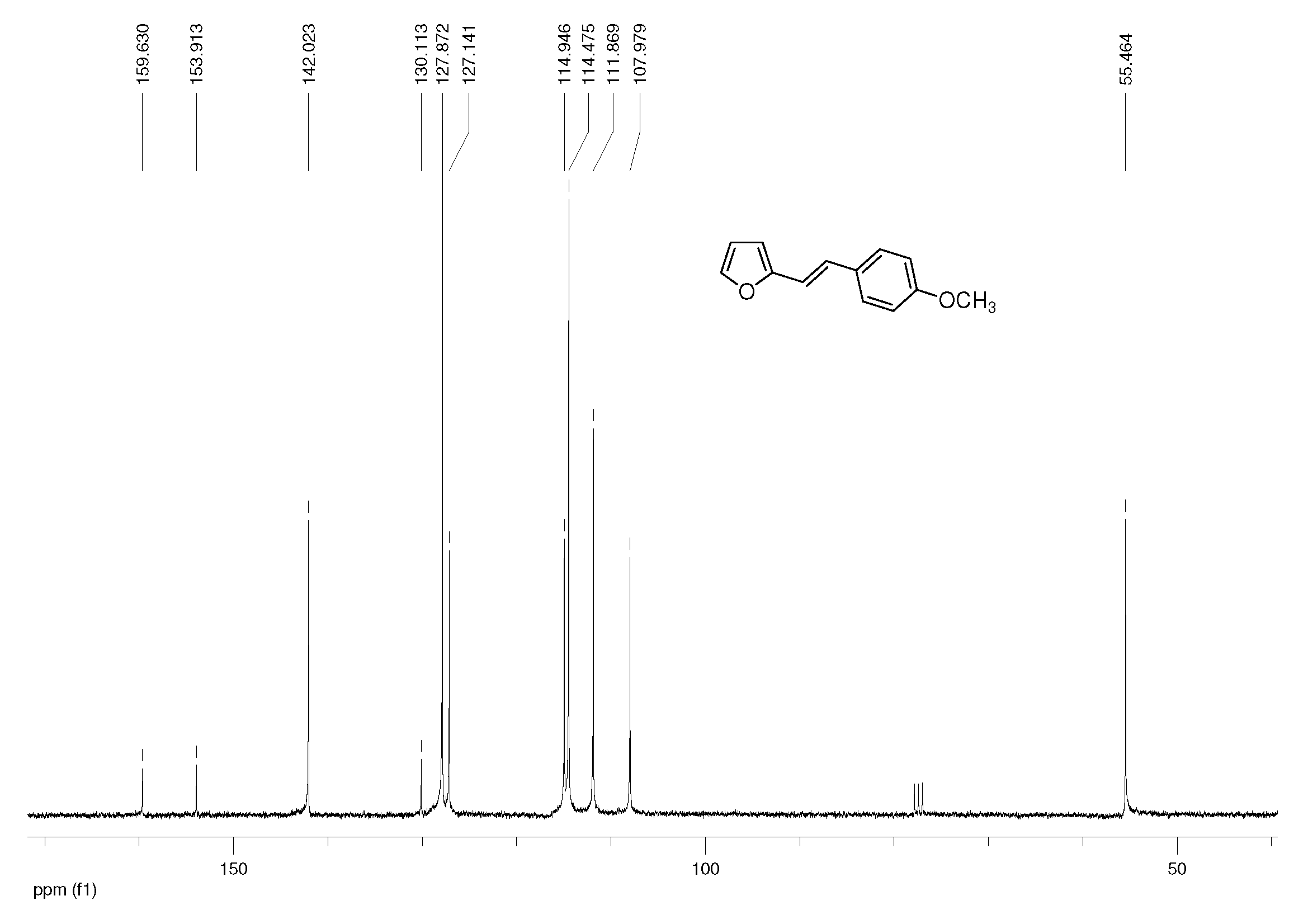

Supplement: Figure S6 — C-NMR of compound 2. (TIF) [file pone.0025421.s006.tif]

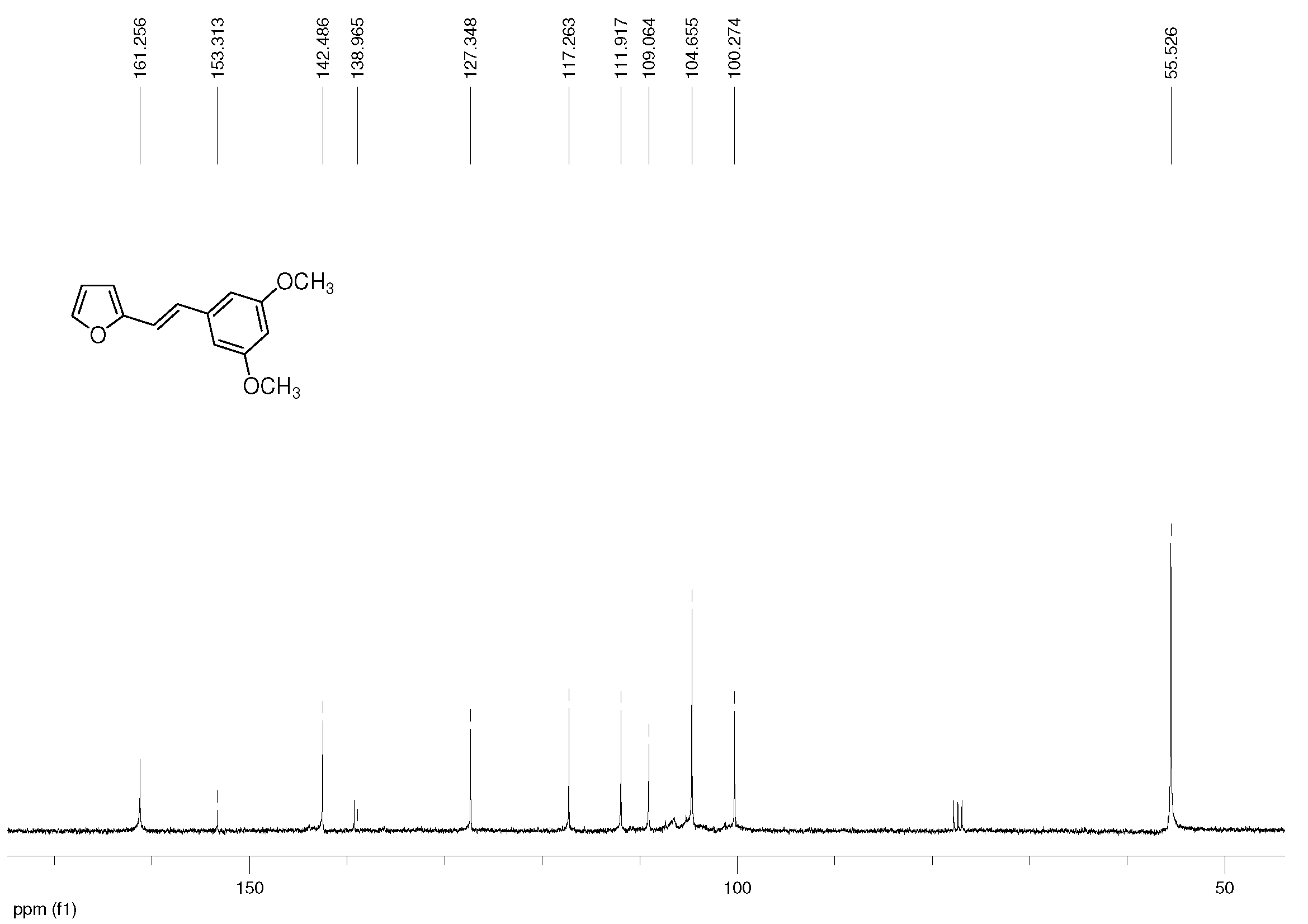

Supplement: Figure S7 — C-NMR of compound 3. (TIF) [file pone.0025421.s007.tif]
